# Systemic lupus erythematosus complicated with cryptococcal meningitis: A case report

**DOI:** 10.1097/MD.0000000000033541

**Published:** 2023-04-14

**Authors:** Honglei Ma, Yuqun Wang, Junhong Liu, Linping Du, Xiaodong Wang, Yingliang Wang

**Affiliations:** a Affiliated Hospital of Weifang Medical University, School of Clinical Medicine, Weifang Medical University, Weifang, People’s Republic of China; b Affiliated Hospital of Weifang Medical University, Weifang, People’s Republic of China.

**Keywords:** central nervous system, cryptococcal, lupus erythematosus, lupus vasculitis, meningitis, systemic

## Abstract

Patients with systemic lupus erythematosus (SLE) complicated with cryptococcal meningitis (CM) are easy to be misdiagnosed as neuropsychiatric lupus or tuberculous meningitis due to the lack of specificity of clinical symptoms, which may delay treatment. Through this case, we considered early improvement of India ink stain of cerebrospinal fluid (CSF) and metagenomic next generation sequences to determine whether there is microbial infection, and gave the idea of empirical anti-infection therapy, so as to make early diagnosis and slow down the progression of the disease.

**Patient concerns:**

We report the case of a 40-year-old female with SLE for 10 years. Five days ago she came down with a fever and a headache.

**Diagnosis, interventions, and outcomes:**

India ink stain of CSF in patients with SLE shows Cryptococcus neoformans growth. Combined with imaging findings, the patient was diagnosed with CM. The patient improved after 3 weeks of antifungal therapy with amphotericin B 42 mg/d and flucytosine 6000 mg/d.

**Lessons:**

The possibility of CM should be considered when SLE patients have sudden headache and fever. India ink stain of CSF and metagenomic next generation sequences should be actively improved in the early stage of the disease to identify whether there is microbial infection, and early empirical anti-infection treatment should be given to reduce mortality.

## 1. Introduction

Systemic lupus erythematosus (SLE) is a multi-organ system autoimmune disease that produces a variety of pathogenic autoantibodies and immune complexes.^[1,2]^ In patients with SLE, 30% to 50% of morbidity and mortality are attributed to infections, mainly of the respiratory and urinary tract, skin and soft tissue, and blood, while central nervous system infections account for only 3% of all infections.^[3,4]^ Cryptococcal meningitis (CM) is the leading cause of death from infectious diseases of the central nervous system worldwide, with about 500,000 people dying from CM every year. The fatality rate is still as high as 25% to 50% after treatment, especially in patients with transplantation or other cellular immune deficiency.^[5,6]^ Patients with SLE complicated with CM are extremely rare. Due to the lack of specificity of clinical symptoms, it is easy to be misdiagnosed as neuropsychiatric lupus and tuberculous meningitis, thus delaying treatment. Therefore, timely diagnosis and effective antifungal treatment are essential. At present, there are few relevant reports in China. This paper reports a case of SLE combined with CM admitted to the Affiliated Hospital of Weifang Medical College.

## 2. Case report

A 40-year-old female was admitted to our hospital with fever for 5 days and headache for 4 days. The patient had a history of SLE for 10 years.

The patient developed a fever 5 days ago, accompanied by chills and sore throat. After taking “Xiaochaihu granules” which is a kind of traditional Chinese medicine, His fever went down. And 4 days ago, the patient had a headache with paroxysmal stabbing pain, unfixed position, unbearable pain, no dizziness. After taking ibuprofen, the headache can be relieved, but the patient still has a recurrent headache, which is full of pain in the head. The pain is unbearable during each attack, lasting from several hours to 1 day, accompanied by intermittent fever. The patient had a 10-year history of SLE, and was routinely treated with “prednisone 10 mg/d, leflunomide 20 mg/d, and hydroxychloroquine 0.4 g/d.”

**Figures 1.**
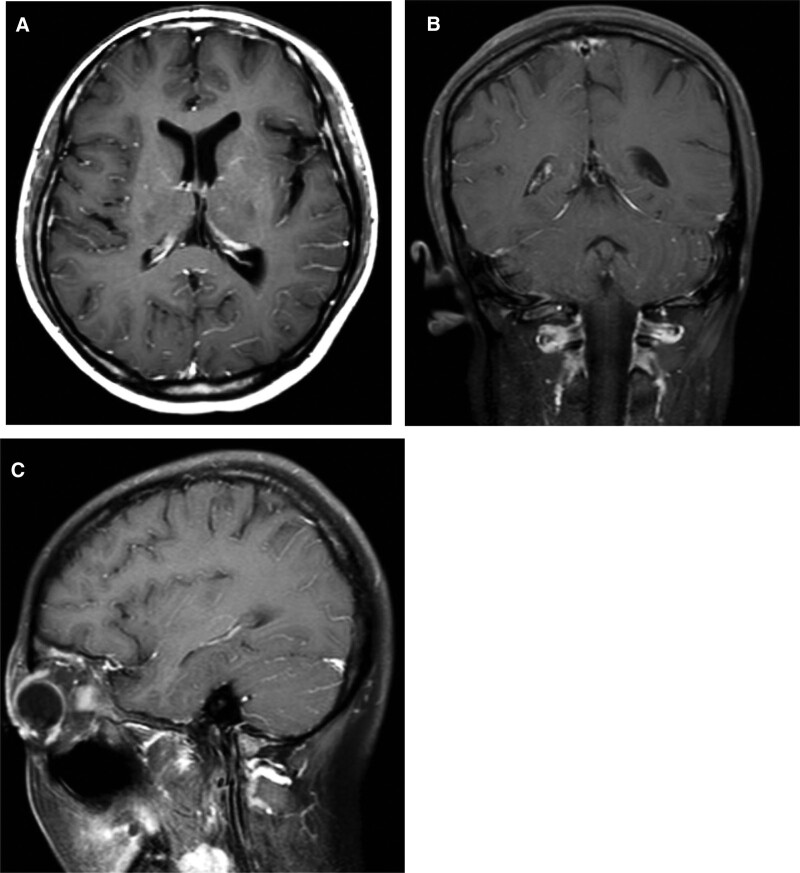
A, B and C show the MRI axial, coronal and sagittal enhancement images on the 10th day of admission, with multiple linear enhancement shadows in the cerebral and cerebellar sulci with smooth margins. MRI = magnetic resonance imaging.

**Figure 2.**
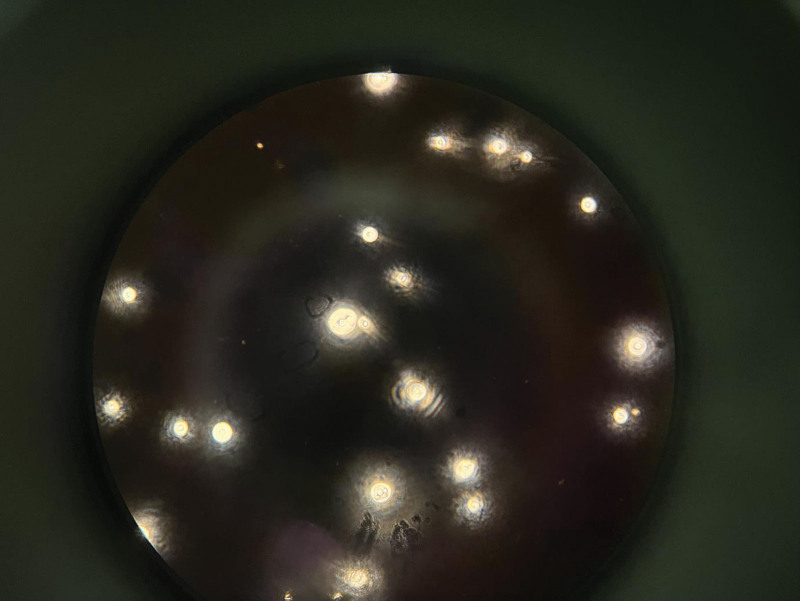
Visible cryptococcal growth (Indian ink stain 400×).

**Figures 3.**
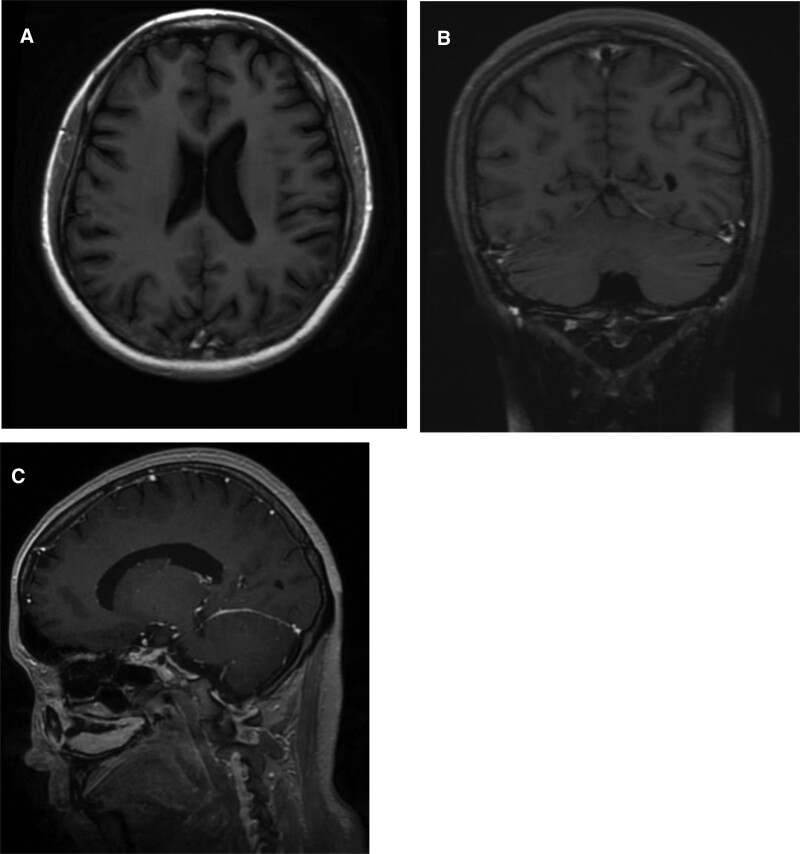
A, B and C show the MRI axial, coronal and sagittal enhancement images after antifungal treatment on the 19th day of admission, with a small amount of enhancement in the cerebellar sulcus and smooth margins. MRI = magnetic resonance imaging.

Her physical examination on admission showed no remarkable findings other than swollen left tonsil and Pharyngeal congestion. There were no neck stiffness, and biceps reflex, triceps reflex and knee reflex were normal, Babinski sign and meningeal stimulation sign were negative. Laboratory examination showed antinuclear antibody + 1:1000 (nuclear particles) (Table 1) and c-reactive protein 26.0 mg/L. No abnormality was found in a variety of fungal tests, rheumatism, and immune function test. Blood routine examination showed white blood cells 8.41*10^9/L, red blood cells 3.31*10^12/L, hemoglobin 87 g/L, and platelets 50*10^9/L (Table 2). Computed tomography of chest showed inflammation in the upper lobe of the left lung, ground glass nodules in the upper lobe of the right lung, and ventricular micronodules in both lungs. Computed tomography of brain showed no obvious abnormalities. Magnetic resonance imaging (MRI) of brain on the 2nd day of admission showed no abnormality in brain parenchyma. Brain enhancement scan showed no significant enhancement of brain parenchyma. Considering the possibility of infection, anti-infection therapy was given with cefazolin 6 g/d, azithromycin 0.5 g/d as anti-infective therapy, and continued prednisone 10 mg/d, leflunomide 20 mg/d, and hydroxychloroquine 0.4 g/d. On the dawn of the 4th day after admission, the patient developed headache and fever again, with the highest temperature reaching 38.0°C, considering that the infection was not controlled, ceftizoxime and azithromycin were stopped and piperacillin-tazobactam 13.5 g/d combined with minocycline 200 mg/d was used for anti-infection treatment. On the early morning of the 5th day after admission, the patient had a headache again, accompanied by nausea and vomiting. Lumbar puncture was performed, initial pressure was 210 mmH_2_O, and cerebrospinal fluid (CSF) analysis showed glucose 2.28 mmol/L. Chlorine tendency for 118.8/L; Protein 0.480 g/L; CSF immunoglobulin G 42.8 mg/L; CSF immunoglobulin M 2.4 mg/L (Table 3). We considered intracranial viral encephalitis, plus acyclovir 1.5 g/d antiviral therapy and oseltamivir 150 mg/d for influenza virus prevention. Fungal spores were detected in the blood culture smear on the 7th day of admission. Considering that there was a fungal infection with low immunity, fluconazole was given 200 mg/d intravenous drip and the hormone was reduced to methylprednisone 40 mg/d. Blood culture (aerobic bacteria) on the 9th day of admission showed candida nameless growth. On the 10th day after admission, the head MRI scan and enhancement showed bilateral brain and cerebellar pia meningeal enhancement, which was consistent with meningitis (Fig. 1). The initial pressure of lumbar puncture CSF was 185 mmH_2_O, and the protein was 0.530 g/L by CSF analysis. CSF fungal D-glucan was detected at 154.50 pg/mL. Fungal spores were detected in the blood culture smear on the 12th day of admission, and fluconazole was given 400 mg/d. Blood culture (aerobic bacteria) on the 13th day of admission showed that cryptococcus neoformation was growing. India ink stain of CSF on day 14 of admission showed the growth of cryptococcus neoformans (Fig. 2). The patient was diagnosed with CM and was given amphotericin B 42 mg/d combined with flucytosine 6000 mg/d antifungal therapy for 3 weeks, and did not develop fever or headache. On January 28, 2022, the reexamination of cranial magnetic resonance plain scan and enhancement showed bilateral cerebral and cerebellar pia meningeal enhancement, which was consistent with meningitis changes and significantly improved compared to the previous (Fig. 3). On February 9, 2022, the lumbar puncture was reexamined, and initial pressure was measured at 150 mmH_2_O. No cryptococcus detected in CSF. Methylprednisolone was discontinued and prednisone was taken orally at 40 mg/d instead. Maintenance therapy of fluconazole 200 mg/d combined with flucytosine 6000 mg/d was continued out of the hospital, and she did not have fever or headache outside the hospital. And the patient’s head MRI has not shown any significant abnormalities on the 2 reviews so far.

**Table 1:** Autoimmune lab results.

Test	Result	Reference range (units)
Antinuclear antibody	+1:1000(speckled pattern)	<1:100
Double-stranded DNA antibody	103 IU/mL	0–120 (IU/mL)
Complement C3	0.96 g/L	0.9–1.8 (g/L)
Complement C4	0.15 g/L	0.1–0.4 (g/L)

**Table 2:** Initial serologic.

Test	Result	Reference range (units)
White blood cell	8.41*10^9^/L	3.5–9.5 (*10^9^/L)
Red blood cell	3.31*10^12^/L	3.8–5.1 (*10^12^/L)
Neutrophile granulocyte	6.60*10^9^/L	1.8–6.3 (*10^9^/L)
Lymphocyte	0.7*10^9^/L	1.1–3.2 (*10^9^/L)
Platelet	50*10^9^/L	125–350 (*10^9^/L)
Eosinophilic granulocyte	0.24*10^9^/L	0.02–0.52 (*10^9^/L)
Basophilic granulocyte	0.01*10^9^/L	0.00–0.06 (*10^9^/L)
C-reactive protein	26.00 mg/L	0–5 (mg/L)
Erythrocyte sedimentation rate	24.0 mm/L	0–20 (mm/L)

**Table 3:** Cerebrospinal fluid studies.

Test	Result			Reference range (units)
	2022/1/14	2022/1/19	2022/2/9	
CellCount	55 × 10^6^/L	40 × 10^6^/L	10 × 10^6^/L	(×10^6^/L)
Protein	0.48 g/L	0.53 g/L	0.14 g/L	0–0.4 (g/L)
Glucose	2.28 mmol/L	3.06 mmol/L	3.97 mmol/L	2.50–4.45 (mmol/L)
Lactate dehydrogenase	15 U/L	12 U/L	16 U/L	10–25 (U/L)
Adenosine deaminase	0.8 U/L	0.3 U/L	0.3 U/L	0–40 (U/L)
Cl	118.8 mmol/L	126.2 mmol/L	128.6 mmol/L	120–130 (mmol/L)
Cerebrospinal fluid-immunoglobulin G	42.8 mg/L	22.3 mg/L	11.9 mg/L	0–34 (mg/L)
Cerebrospinal fluid-immunoglobulin M	2.4 mg/L	0.9 mg/L	0.2 mg/L	0–1.3 (mg/L)
Cerebrospinal fluid-immunoglobulin A	5 mg/L	1.1 mg/L	1.7 mg/L	0–5 (mg/L)
India ink stain	No bacteria	Cryptococcus neoformans	No bacteria	

## 3. Discussion

SLE is a systemic autoimmune disease involving multiple systems in which the immune system attacks healthy cells and tissues throughout the body.^[7,8]^ CM is the most common cryptococcus infecting humans and is usually spread through bird droppings, plants, dust, soil, and contaminated food.^[9,10]^ The specific mechanism of SLE with CM is unclear, but hormone induction of cellular immune deficiency through several complex immunosuppressive effects, combined with immunosuppressive agents, leads to increased susceptibility to invasive fungi and mortality.^[11]^ In addition, immune abnormalities associated with SLE, such as immunoglobulin deficiency, complement deficiency, and reduced complement receptor expression, increase the likelihood of developing opportunistic infections, especially fungal infections.^[12]^ Thus, SLE patients are vulnerable to cryptococcus.

In addition, we need to discuss drug-induced aseptic meningitis (DIAM). There are 4 main classes of drugs associated with aseptic meningitis: non-steroidal anti-inflammatory drugs, especially ibuprofen, are associated with an increased risk in patients with SLE; antibiotics; intravenous immunoglobulin; monoclonal antibodies.^[13]^ The patient had been treated with ibuprofen. DIAM may be due to 2 mechanisms: a direct meningeal irritation caused by the intrathecal administration of drugs and an immunologic hypersensitivity reaction to a systemic administration. If it is a direct meningeal stimulation it is fairly easy to recognize, whereas immune hypersensitivity is difficult to recognize. DIAM associated with systemic therapy usually develops early in the week, a period that can be reduced to a few hours in case the medication is challenged again, and symptoms caused by ibuprofen (fever, chills, headache, vomiting, joint pain, generalized myalgia, rash, stiff neck, abdominal pain, and confusion) usually appear hours after taking the medication.^[14,15]^ After discontinuation of the suspected drug, the clinical symptoms will resolve quickly and spontaneously. Other than these temporal references, there are no specific clinical or biological parameters.^[14]^ Although the patient in this case had a history of SLE, the history of using ibuprofen was 4 days after the patient had headache and fever, and the symptoms were relieved after the application. The subsequent cultivation of cryptococcus in CSF ruled out the diagnosis of aseptic meningitis caused by ibuprofen.

The patient’s history, symptoms, signs, and examination results had the following characteristics. First, the patient presented with fever and headache and had a history of thrombocytopenia, so we first considered the possibility of cerebral hemorrhage, but computer tomography scan of brain helped us rule out cerebral hemorrhage. Second, the patient had a history of SLE for 10 years and had been treated with “prednisone 10 mg/d, leflunomide 20 mg/d, and hydroxychloroquine 0.4 g/d” for a long time. The sudden onset of central nervous system symptoms did not rule out the possibility of lupus encephalopathy and tuberculous meningitis. Cryptococcal growth was detected by subsequent lumbar puncture and ink staining of CSF, and the diagnosis of CM was finally confirmed. In fact, when the doctor first proposed lumbar puncture for CSF testing, the patient expressed her refusal, believing that she only had a headache caused by SLE and that it could not be meningitis caused by pathogenic infection. We educated the patient about the relevant medical knowledge and informed him that the application of immunosuppressants for SLE greatly increases the risk of infection, and the patient eventually agreed to the lumbar puncture. After the first CSF results suggested no pathogens and no abnormalities on imaging, but the patient still had persistent fever and headache, we suggested the patient to have another lumbar puncture for CSF testing, which he again refused. After we emphasized the need for CSF testing, the patient agreed. And This time, novel cryptococci were detected in the CSF. Unfortunately, if we had performed metagenomic next generation sequences (mNGS) on the first CSF test, it is likely that we would have been able to detect cryptococcal infection and thus confirm the diagnosis on the first try. After diagnosis, fluconazole 800 mg/d combined with flucytosine 6000 mg/d was given antifungal therapy for 3 weeks. No cryptococcus was found in CSF after 16 days of treatment. Out-of-hospital consolidation treatment: Fluconazole 200 mg/d combined with flucytosine 6000 mg/d for maintenance treatment, which is still continuing at present, and no obvious abnormality was found in cranial magnetic resonance examination twice during the period. Therefore, in SLE patients with unexplained headache and other neurological symptoms, we should think out of the box and consider whether the patient is complicated by novel CM. Early refinement of India ink stain or mNGS of CSF to clarify the presence of pathogenic infection is essential.^[16,17]^

Patients with CM perform lumbar punctures and often show an abnormal increase in intracranial pressure. An abnormal increase of intracranial pressure can cause severe headache, vomiting, and even consciousness disorder, dilated pupil, heartbeat, and respiratory arrest. Intracranial hypertension is associated with 40% of deaths within 3 to 10 weeks of CM, and the longer a patient is misdiagnosed, the worse the prognosis.^[18]^ Timely and effective control of intracranial hypertension is one of the most critical factors to determine the outcome of CM.^[19]^ In 2010, the Infectious Diseases Society of America revised guidelines stating that if there is an increase in CSF pressure ≥ 25 mmH_2_O during induction therapy and there are symptoms of increased intracranial pressure, CSF drainage (which reduces the open pressure of the CSF by 50% or to normal pressure) is used, and mannitol is unproven and not routinely recommended.^[20]^

This is a case of a patient with SLE who suddenly has a fever and a headache. Novel cryptococcal infection detected after multiple India ink stain of CSF. Meanwhile, cerebral magnetic resonance imaging showed meningitis. Therefore, the possibility of cryptococcus neoformans infection, early CSF test or the improvement of mNGS should be considered in SLE patients with long-term use of immunosuppressants.

## Acknowledgments

We acknowledge Rheumatology and Immunology department, Affiliated Hospital of Weifang Medical University, Scientific research project of Weifang Health Commission, China (WFWSJK-2022-015) and Shandong Provincial Natural Science Foundation, China (ZR2017LH039).

## Author contributions

**Conceptualization:** Honglei Ma, Yingliang Wang.

**Data curation:** Honglei Ma, Junhong Liu.

**Investigation:** Yuqun Wang, Linping Du.

**Supervision:** Xiaodong Wang, Yingliang Wang.

**Writing – original draft:** Honglei Ma.

**Writing – review & editing:** Yingliang Wang.
